# A Cerebral Organoid Connectivity Apparatus to Model Neuronal Tract Circuitry

**DOI:** 10.3390/mi12121574

**Published:** 2021-12-17

**Authors:** Denise A. Robles, Andrew J. Boreland, Zhiping P. Pang, Jeffrey D. Zahn

**Affiliations:** 1 Department of Biomedical Engineering, Rutgers University, 599 Taylor Road, Piscataway, NJ 08854, USA; denise.robles@rutgers.edu; 2 Child Health Institute of New Jersey, Robert Wood Johnson Medical School, 89 French Street, New Brunswick, NJ 08901, USA; andrew.boreland@rutgers.edu (A.J.B.); pangzh@rwjms.rutgers.edu (Z.P.P.); 3 Department of Neuroscience and Cell Biology, Robert Wood Johnson Medical School, 675 Hoes Lane West, Piscataway, NJ 08854, USA; 4 Pediatrics, Robert Wood Johnson Medical School, Rutgers University, One Robert Wood Johnson Place, MEB, New Brunswick, NJ 08903, USA

**Keywords:** cerebral organoid, microfabrication, microfluidics, organ-on-a-chip, cerebral tract, neuronal culture, organotypic slice

## Abstract

Mental disorders have high prevalence, but the efficacy of existing therapeutics is limited, in part, because the pathogenic mechanisms remain enigmatic. Current models of neural circuitry include animal models and post-mortem brain tissue, which have allowed enormous progress in understanding the pathophysiology of mental disorders. However, these models limit the ability to assess the functional alterations in short-range and long-range network connectivity between brain regions that are implicated in many mental disorders, e.g., schizophrenia and autism spectrum disorders. This work addresses these limitations by developing an in vitro model of the human brain that models the in vivo cerebral tract environment. In this study, microfabrication and stem cell differentiation techniques were combined to develop an in vitro cerebral tract model that anchors human induced pluripotent stem cell-derived cerebral organoids (COs) and provides a scaffold to promote the formation of a functional connecting neuronal tract. Two designs of a Cerebral Organoid Connectivity Apparatus (COCA) were fabricated using SU-8 photoresist. The first design contains a series of spikes which anchor the CO to the COCA (spiked design), whereas the second design contains flat supporting structures with open holes in a grid pattern to anchor the organoids (grid design); both designs allow effective media exchange. Morphological and functional analyses reveal the expression of key neuronal markers as well as functional activity and signal propagation along cerebral tracts connecting CO pairs. The reported in vitro models enable the investigation of critical neural circuitry involved in neurodevelopmental processes and has the potential to help devise personalized and targeted therapeutic strategies.

## 1. Introduction

Mental disorders are the leading cause of disability and the second leading cause of death worldwide [1,2]. The global burden has increased significantly over the past few decades and is expected to continue rising due to prolonged life expectancy. A 2019 report estimated approximately 20.6% of adults in the United States have a mental illness, with 5.2% having a serious mental illness which severely impacts their quality of life [3]. Within the human brain, functional neural circuits consist of neuronal axons which extend to integrate different brain regions and create robust cerebral tracts. These white matter tracts mediate intercommunication between brain areas and enable information transfer critical for brain function. Evidence suggests that deficits in short-range and long-range circuitry connecting brain regions are implicated in neuropsychiatric disorders such as autism spectrum disorders [4,5,6] and schizophrenia [7,8,9]. Due to the vast complexity of the human brain and limited accessibility to live viable tissue, a cerebral tract platform which closely recapitulates human brain tissue on the cellular and cytoarchitectural levels would provide a valuable tool for researchers investigating dysfunction in mental disorders. This work combines stem cell organoid technology with microfabricated culture systems to develop reliable models of human brain circuitry to facilitate efficient development and clinical translation of therapeutic treatments of mental disorders.

Microfabrication has emerged as a promising tool for the fabrication of compartmentalized in vitro brain-on-a-chip (BoC) systems of human neurocircuitry, where microchannel arrays segregate single axons from neuronal cell bodies. Several groups have reported the fabrication of compartmentalized two-dimensional (2D) cell culture platforms in modeling functional neural circuits and their utility in studying neuronal communication deficits [10,11,12,13]. Recent advances in human stem cell biology have furthered the potential of these systems to model physiologically relevant human brain circuitry. Our group recently reported a compartmentalized culture model using induced neuronal cells derived from human induced pluripotent stem cells (hiPSCs) for use in high-throughput pharmacological screening assays [14]. While these 2D compartmentalized systems are useful for modeling axonal injury and neuronal communication at the single-axon level, they do not accurately reflect the complex three-dimensional (3D) in vivo brain environment in terms of cytoarchitecture and input from multiple cell types. Emerging BoCs are designed to recapitulate the native brain architecture by engineering 3D cerebral systems in microfabricated platforms, which can provide an extrinsic microenvironment reminiscent of the in vivo state [15].

Cerebral organoids (COs) derived from hiPSCs have emerged as a promising tool for modeling functional 3D human neural tissue. COs spontaneously self-assemble and mature into a 3D neural architecture [16], providing a unique opportunity to model mental disorders in a patient-specific context. COs have the intrinsic ability to generate diverse functional neuronal and glial subtypes which exist in vivo, including glutamatergic neurons, dopaminergic neurons, GABAergic neurons, and oligodendrocytes [17,18]. Stem-cell derived COs also share proteomic similarities with fetal human brain tissue [19,20,21], highlighting their distinct potential to recapitulate early human brain development and formation of neurocircuitry. COs can also be patterned toward specific brain region lineages [22,23,24,25] that when combined in a multi-organoid platform, enables the development of more complex neurocircuitry models. Existing 3D neural tissue models of long-range connectivity either do not permit long-term culturing periods due to a lack of recirculating flow and spatial constraints [26] or do not demonstrate reliable functional connectivity properties [27]. Thus, there is an unmet need for an in vitro model system of long-range connectivity using hiPSCs which addresses limitations in structural organization, long-term survival [26], human specificity, and functional connectivity [27].

To further improve on these technologies, this research focuses on the development of a model system composed of SU-8 photoresist structures designed to anchor COs at a defined distance and promote the formation of an inter-organoid cerebral tract, termed the Cerebral Organoid Connectivity Apparatus (COCA). This report details the fabrication and utility of two COCA versions to accommodate the foremost CO study paradigms: the first design contains a series of spikes in the CO-anchoring area which secure the organoid (spiked COCA), while the second design contains supporting structures onto which COs are attached (grid COCA). These designs also allow effective media exchange around the CO surface. Both designs contain two regions for attaching COs separated by a scaffold track which promotes axonal extension between both sides to create a cerebral tract. The COCA is also accessible and amenable to live-track imaging and electrophysiological characterization. In this work, COCA systems were used to evaluate the morphology and functional intracellular signaling of CO cultures and connecting tracts using fluorescent microscopy, calcium imaging, and viral tracing techniques. Results illustrate the formation of bidirectional long-range neuronal tracts between CO cultures and functional signaling dynamics within CO bodies and along the connecting tracts, demonstrating the potential of this system to model human neurocircuitry and advance treatment development for disorders associated with disrupted neural tract connectivity.

## 2. Materials and Methods

The COCA design centers on compartmentalizing CO pairs and forming functional neuronal connections between each side to mimic in vivo cerebral tract circuitry. Two COCA versions were developed: the spiked COCA design features spiked structures in each ring-shaped compartment which anchor the COs in place, and a connecting channel which joins the two compartments to enable neurite extension; the grid COCA design features an open grid structure which permits media flow along the CO surface to improve nutrient diffusion and prolong long-term viability. The devices and fluidic culturing platform were fabricated using long-wave UV (LWUV) light photolithography of SU-8 photoresist and soft lithography techniques, respectively.

### 2.1. Fabrication of the COCA

**Figure 1:**
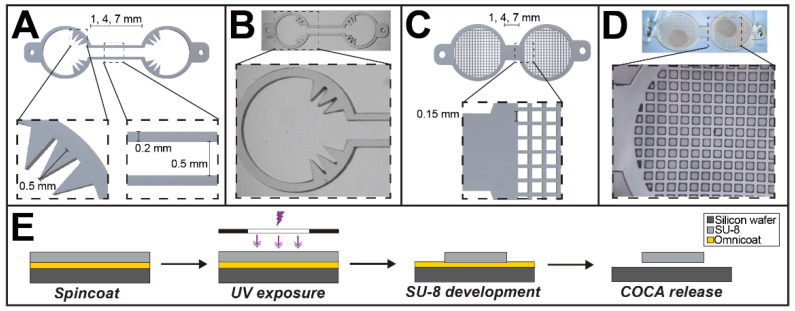
Design and fabrication of the spiked or grid Cerebral Organoid Connectivity Apparatus (COCA). (**A**) Computer-aided design (CAD) rendering of the spiked COCA design, featuring two spiked cerebral organoid (CO)-anchoring ring-shaped compartments connected by a joining channel. (**B**) Brightfield microscope images of a fabricated spiked COCA. Two tabs were designed on either side to support placement of the COCA within a perfusion chamber. (**C**) CAD rendering of the grid COCA design, featuring two grid compartments for CO slice culture. (**D**) Brightfield microscope images of a fabricated grid COCA with a CO slice culture. (**E**) Overview of the major microfabrication steps involved in fabricating COCAs using a transparency mask and photolithography techniques.

Both the spiked and grid COCA devices were designed using AutoCAD modeling software (Autodesk, Inc., Version 2020, Mill Valley, CA, USA) and printed as transparency masks for photolithography (CAD/Art Services, Inc., Bandon, OR, USA). A robust lift-off protocol was developed to release the SU-8 photoresist COCA structures from silicon wafers (Figure 1). SU-8 is an epoxy-based negative photoresist commonly used for microfabrication due to its mechanical and chemical stability, and high-aspect ratio properties which enable precise definition of sub-micron features. Briefly, a four-inch silicon wafer was cleaned with acetone, isopropyl alcohol (IPA), and deionized water for 10 min each and baked at 150 °C for a minimum of 30 min. A first layer of Omnicoat (Kayaku Advanced Materials, Inc., Westborough, MA, USA) was spun onto the wafer at 3000 revolutions per minute (rpm) to achieve a thickness of approximately 13 nm and baked at 200 °C for 1 min. This thin coating allows for the release of the SU-8 structures from the wafer at the end of the process. In order to achieve a device thickness of approximately 320 μm, a combination of two SU-8 photoresists (Kayaku Advanced Materials, Inc.) were used. SU-8 2075 was spun onto the wafer at 1000 rpm, soft-baked at 65 °C for 10 min and 95 °C for 50 min and allowed to cool to room temperature for one hour. Next, SU-8 2025 was spun and baked using the same procedure. The wafer was exposed to UV light using a transparency mask of the device features, baked at 65 °C for 5 min and 95 °C for 15 min, and subsequently developed using SU-8 developer on an orbital shaker at 60 rpm to remove unexposed regions of the photoresist. After rinsing with IPA and drying using a compressed air gun, MF-319 developer was used to remove the underlying Omnicoat, enabling release of the SU-8 structures. After a hard bake of 200 °C for 30 min, the devices were extensively cleaned with IPA and deionized water. Fabrication of each batch of COCAs was performed using strict procedures and devices were examined via microscopy between each step to ensure proper definition of SU-8 structures and material properties. Optimization of the fabrication procedure revealed that slow hard-baking during the SU-8 photoresist patterning steps and a final hard-baking step were found to be critical to anneal microcracks and undulations on the SU-8 surface. Prior to cell culture, COCAs were sterilized with UV light in a culture hood for 30 min. A summary of the main design parameters of the two COCA versions is shown in Table 1. 

### 2.2. Cerebral Organoid Generation

COs were generated using published protocols [16,18,28,29]. Briefly, hiPSC embryoid bodies were induced toward a neuronal fate using small molecules in neural induction medium consisting of noggin (50 ng/mL, Peprotech, East Windsor, NJ, USA), SB431542 (5 μM, Stemgent, Beltsville, MD, USA), DMEM/F12 (HyClone, Logan, UT, USA), and 1× N2 (Thermo Fisher Scientific, Waltham, MA, USA) for 1 week. The EBs were then plated on growth-factor reduced matrigel for 1 week in DMEM/F12 (HyClone), and 1× N2 (Thermo Fisher Scientific) with 10 μg/mL Laminin (Sigma-Aldrich, St. Louis, MA, USA). After 1–2 weeks of culture, neural rosettes were manually isolated and further cultured to produce a pure neural progenitor cell (NPC) population in pNPC media consisting of 1× N2 (Thermo Fisher Scientific), 1× B27 minus vitamin A, bFGF (20 ng/mL, Peprotech), SB431542 (2 μM, Stemgent), human leukemia inhibitory factor (10 ng/mL, Peprotech), CHIR99021 (3 mM, STEMCELL Technologies Inc., Vancouver, BC, Canada), and ROCK inhibitor Y-27632 (10 mM, Tocris Bioscience, Bristol, UK) in a 1:1 ratio of DMEM/F12 (Hyclone) and neurobasal medium (Thermo Fisher Scientific). Organoids were generated by plating approximately 10,000 NPCs per well in a low-adherence 96-well plate and culturing for one week in NPC medium consisting of 1× N2 (Thermo Fisher Scientific), 1× B27 minus vitamin A, and bFGF (20 ng/mL, Peprotech) in a 1:1 ratio of DMEM/F12 (Hyclone) and neurobasal medium (Thermo Fisher Scientific). The organoids were then moved into a low-adherence six-well plates (Corning Inc., Corning, NY, USA) containing neuronal differentiation (ND) medium composed of 1:1 ratio of DMEM/F12 (Hyclone) and neurobasal medium (Thermo Fisher Scientific) supplemented with 1× N2, 1× B27, 10 ng/mL GDNF (Peprotech), 10 ng/mL BDNF (Peprotech), 1 μM Dibutyryl-cAMP (STEMCELL Technologies Inc., Vancouver, BC, Canada), and 200 μM L-ascorbic acid (Sigma-Aldrich). Half medium changes were performed every 2 days with COs cultured on an orbital shaker at 110 RPM until used for COCA plating. For the grid COCA experiments, COs were vibratome sliced to a 500 μm thickness at day 97 and immediately placed onto either side of the device.

### 2.3. CO Immunohistochemistry

**Figure 2:**
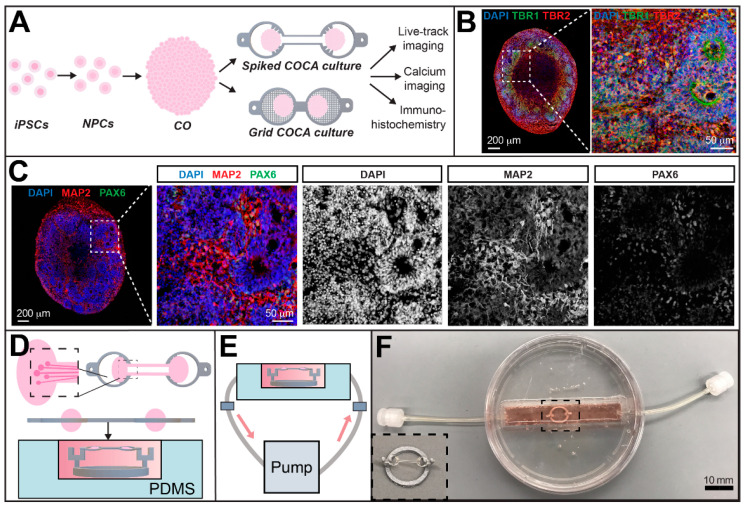
Overview of culturing protocol and perfusion chamber. (**A**) Overview timeline of CO generation and device culturing procedure, starting from human induced pluripotent stem cells. The generated organoids are placed onto the spiked or grid COCA to support the formation of a connecting cerebral tract between ends. The open-top culturing system enables the use of standard characterization metrics for neuronal cultures. (**B**) Representative immunostaining of CO (Day 97) showing DAPI nuclear counterstain, and expression of TBR1 and TBR2. (**C**) Representative immunostaining of CO (Day 97) showing DAPI (4′,6-diamidino-2-phenylindole) staining and expression of neuronal markers MAP2 and PAX6. (**D**) Schematic representation of the spiked COCA with cultured COs and connecting cerebral tract. (**E**) Schematic of perfusion chamber system including a peristaltic pump, silicone tubing, tubing connectors, polydimethylsiloxane (PDMS) chamber, Polylactic acid (PLA) stand, cell culture media, and a COCA culture. (**F**) Photograph of a perfusion chamber containing media, PLA stand, and COCA. Inset shows a zoomed-in image of the PLA stand and COCA.

After CO generation, they were fixed, sectioned, and processed for immunofluorescence staining using previously reported protocols (Figure 2B,C) [29,30,31,32]. Briefly, organoids were fixed in 4% paraformaldehyde, cyroprotected in 15% and 30% sucrose, embedded in OCT freezing medium, snap-frozen, and sectioned at 10–20 μm using a cryostat. The primary antibodies used for immunohistochemistry were PAX6 (rabbit, 1:250 dilution, Novus Biologicals Cat. #NB300-750, Littleton, CO, USA), MAP2 (chicken, 1:1000 dilution, MilliporeSigma Cat. #AB5543, Burlington, MA, USA), TBR1 (rabbit, 1:500 dilution, Abcam Cat. #AB31940, Cambridge, UK), and TBR2 (chicken, 1:200 dilution, MilliporeSigma Cat. #AB15894). Alexa Fluor secondary antibodies were used at a 1:500 dilution. Slides were mounted using Fluoromount G medium containing DAPI (4′,6-diamidino-2-phenylindole, SouthernBiotech, AL, USA). Images were obtained using a Zeiss 710 confocal microscope (Zeiss, Oberkochen, Germany). Fluorescent micrographs were processed using image processing software Fiji (NIH ImageJ, Version 1.53h, Bethesda, MD, USA).

### 2.4. CO Culturing on the COCA

The COCA surface was treated with Matrigel (Corning Inc., 1:25 ratio in DMEM) and incubated at 37 °C in a humidified CO_2_ incubator for 2 h. The COCA was transferred into a dish containing ND medium and COs. COs were carefully embedded on the spikes in the spiked COCA to hold them firmly in place (Figure 2A). For the grid COCA cultures, a CO slice was gently placed onto each grid compartment. The COCAs were incubated at 37 °C and 5% CO_2_ in static conditions for approximately two days before transferring to the fluidic chamber with recirculating flow (Figure 2D–F). Images of CO morphology and neurite growth over time were acquired using an EVOS inverted light microscope (Thermo Fisher Scientific).

### 2.5. CO Culture in a Perfusion Chamber

A perfusion chamber for CO culturing was fabricated using soft lithography techniques. A 3D printed block (75 mm × 12.5 mm × 15 mm) was placed in the center of a tissue culture plastic dish (100 mm × 15 mm). A polydimethylsiloxane (PDMS) mixture at a 1:10 ratio of elastomeric agent and elastomeric base (Ellsworth Adhesives Cat. # 2065622, Germantown, WI, USA) was poured into the dish to a height of approximately 10 mm and desiccated for 15 min until all bubbles were removed. The PDMS was cured at 60 °C for at least 1 h before removing the block. An inlet and outlet were drilled on either side of the dish and PDMS using a 1/8” drill bit. The inlet and outlet of the chamber were connected to Tygon silicone tubing (ID 1/32”, VWR International, Radnor, PA, USA) and the tubing was connected to a peristaltic pump (Baoding Lead Fluid Technology Co., Ltd. Cat. #BQ80S, Baoding, China). A polylactic acid stand was 3D printed using an Ultimaker 3 (Ultimaker, Utrecht, The Netherlands) and placed in the center of the chamber. Either the spiked or grid COCA device was placed on the stand via the support tabs and held in suspension in order to allow media to flow along all sides of the tissue. Prior to cell culturing, the PDMS chamber in the dish was sterilized under UV light in a culture hood. The PDMS chamber and tubing were washed thrice with both 70% ethanol and Dulbecco’s Phosphate Buffered Saline (DPBS, Sigma-Aldrich). The PDMS chamber filled with solution was maintained inside of an incubator at 37 °C and 5% CO_2_ while the peristaltic pump was placed a short distance outside of the incubator. The inlet and outlet tubing spanned between the inside and outside of the incubator to permit/establish a closed loop of recirculating flow (Figure 2E,F).

After 2 days in static culture, the COCA was transferred to the perfusion chamber. Before COCA placement, the chamber and connected tubing were filled with ND medium. Tubing end caps at either end of the tubing kept the solution contained to avoid spillage and reduce the risk of contamination during transport between the incubator and the culture hood. After transferring the COCA culture to the fluidic platform, the media was perfused using the peristaltic pump at a flow rate of 3 mL/min. Fifty percent of the media was changed every 3–4 days.

### 2.6. Calcium Imaging of COs on the COCA

Calcium imaging was performed on a CO pair (64 DIV) that had been cultured on a spiked COCA for approximately 16 days. Calcium signaling was measured using calcium indicator Fluo-4AM (ThermoFisher Scientific) and imaged at 820 nm using a two-photon microscope (Scientifica, Uckfield, UK). Briefly, the COCA culture was incubated in a loading buffer solution containing Fluo-4AM (5 μM, Molecular Probes, Eugene, OR, USA) and 0.04% Pluronic F-127 at 37 °C for 45 min on an orbital shaker. The COCA culture was then transferred into fresh neuronal differentiation media for at least 30 min before transferring to a submersion-type recording chamber (Warner Instruments, Inc., Holliston, MA, USA) perfused at room temperature with artificial cerebral spinal fluid solution (125 mM NaCl, 2.5 mM KCl, 1.25 mM NaH_2_PO_4_, 25 mM NaHCO_3_, 2.5 mM CaCl_2_, 1.2 mM MgCl_2_) pH 7.4 bubbled with 5% CO_2_, 95% O_2_. After recording baseline activity, potassium chloride (80 mM, KCl) solution was added to the chamber and the resulting fluorescent intensity was recorded. KCl is known to reliably depolarize neurons, causing a calcium ion influx into functional neurons. Spontaneous and induced electrical activity within each CO on the COCA and along the connecting channel was measured using an Olympus IX81 inverted microscope (Olympus Corporation, Tokyo, Japan) and 20× water immersion objective. Time-lapse imaging was performed every 1 s for 5 min and was collected at 512 × 52-pixel resolution. Image analysis was performed using Fiji software (NIH ImageJ). Regions of interest (ROIs) were placed around the soma and/or connecting fiber tracts. Fluorescence was expressed as relative fluorescence changes (∆F/F) after background subtraction from a neighboring region.

### 2.7. AAV Transduction of COs

COs were transduced with either enhanced green fluorescent protein (eGFP) or mCherry adeno-associated viruses (AAVs) to distinguish their respective projections and innervations. Lipofectamine 2000 reagent (ThermoFisher Scientific Cat. #STEM00001) in Opti-MEM I Reduced Serum Media was used to transduce the neural progenitor cells at a concentration between 100,000 and 200,000 cells per well of a 24 well-plate. Briefly, 0.75 μg of DNA was diluted in 50 μL OptiMEM and 0.75 μL Lipofectamine 2000 was diluted in 50 μL Opti-MEM. Both volumes were mixed and incubated at room temperature for 5 min. The mixture was added to 100 μL of cell suspension, incubated at room temperature for 1 h, and dispensed into a well. The media was replaced the next day and the culture was visualized for fluorescence using a fluorescent microscope (EVOS).

## 3. Results

### 3.1. Fabrication of the COCA

The COCA was fabricated using conventional photolithography techniques. The overall geometry and corresponding dimensions for both spiked and grid COCA versions is shown in Figure 1A–D. Microfabrication of the COCAs can yield over 30 devices through the processing of a single four-inch silicon wafer depending on overall placement of features on the printed transparency mask. The described fabrication protocol involving two SU-8 layers and subsequent release from the wafer is a robust procedure that resulted in little variation between different wafer batches. Moreover, the peristaltic pump-based perfusion platform provides continuous recirculating media flow to the CO, enabling long-term culture and providing a cost-efficient alternative to orbital shakers or bioreactors commonly used for maintaining organoid cultures.

### 3.2. Cerebral Tract Formation between COs on the Spiked COCA

**Figure 3:**
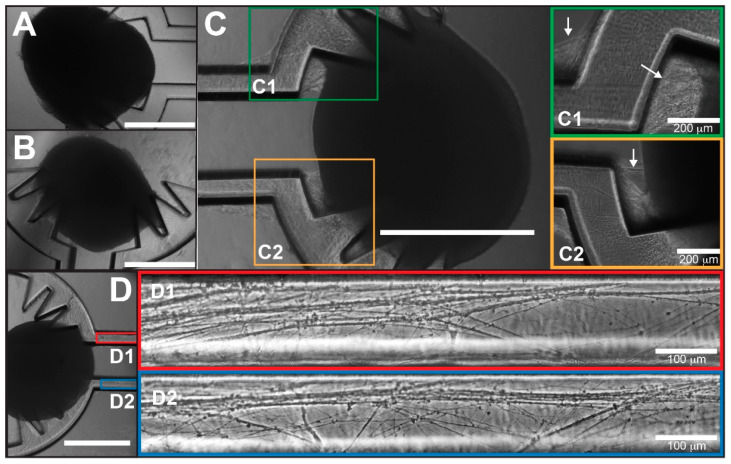
Neurite outgrowth and formation of neurite tract between CO cultures on spiked COCA. (**A**) and (**B**) show brightfield images of CO attachment on the COCA on day 0 post-placement. Brightfield images after 10 days in culture revealed (**C**) neurite bundle formation, (**C1** and **C2** insets) zoomed in images showing detailed neurite bundle formation indicated by arrows, and (**D**) neurite extension along the COCA channel where (**D1** and **D2** insets) show individual axon growth along the COCA channel. Scale bar: 1000 μm unless otherwise noted.

COs were generated from human iPSCs following the neural induction culturing protocol outlined in Figure 2A. Immunohistochemistry analyses of organoid slices revealed robust expression of mature dendritic marker MAP2, cortical forebrain marker PAX6, and cortical neuronal subtype markers TBR1 and TBR2, confirming neuronal identity and cellular viability (Figure 2B,C). After each CO body or slice was gently anchored onto a spiked or grid COCA compartment, it was determined that incubating in static flow conditions for approximately two days allowed sufficient time for attachment of the tissue to the substrate surface before transferring into the fluidic platform shown in Figure 2D–F. Within two days post-placement, the formation of robust neurite bundle structures between each CO and their respective COCA compartment could be observed (Figure 3A,B). There was extensive neurite growth and cellular migration on the surrounding ring structures as well as on the connecting channel edges (Figure 3C,D). This showed that the compartments sufficiently anchored the COs to the COCA under continuous fluidic perfusion. After only three days in culture, single CO neurites from both sides had bridged half of the 5 mm distance along the channel toward the adjacent compartment (Figure 3D). Longer culturing periods resulted in the longitudinal neurites bundling together to form robust neurite fascicles along the connecting channel. The device areas surrounding each CO displayed the highest cellular density, which facilitated gradual conformation of the COs to the shape of the device. These observations demonstrated that the design geometry and functionalized surface of the spiked or grid COCA promote neurite outgrowth from the COs and consequent formation of neuronal tracts along the channel.

### 3.3. Calcium Imaging Reveals Functional Intracellular Signaling

**Figure 4:**
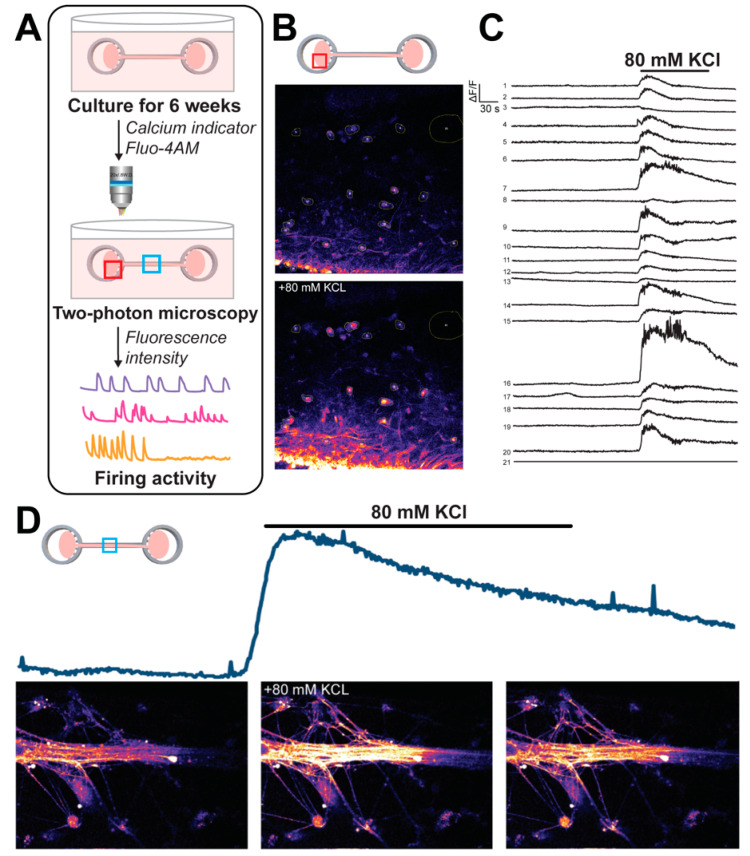
Functional intracellular activity of CO pairs on COCA. (**A**) Schematic of calcium imaging experimental approach performed on day 64 COs. (**B**) Two-photon microscope images of spontaneous and KCl-induced calcium signaling in CO neurons with (**C**) representative cell traces at baseline and after KCl addition. (**D**) Intracellular calcium ion release activity and representative images in an axonal tract at baseline (0 s), immediately after KCl addition (7 s), and at the end of the recording (22 s).

In order to assess functional activity in connected CO pairs (day 64), calcium imaging was performed using a calcium indicator Fluo-4AM. Figure 4A shows the workflow in which COs were cultured on the spiked COCA for several weeks, incubated with the calcium dye, and imaged for fluorescent signaling via two-photon microscopy. Measured fluorescent intensity values indicate the relative level of intracellular calcium ion release activity presented by a particular cell or neurite. Spontaneous, synchronized events were observed in each CO as well as in the neuronal fiber tract connecting both COs. After recording baseline signals, 80 mM KCl was added to the electrophysiology chamber, eliciting a significant increase in activity relative to baseline across all measured regions. Cells on the outer surface of the CO body were most influenced by the KCl addition, likely due to their proximity to the surrounding KCl stimulus (Figure 4B). After the addition of KCl, the traces of cell body fluorescent intensities revealed synchronized calcium dynamics across all visible cells (Figure 4C). A similar phenomenon was observed for cell bodies and neurites located along the COCA channel (Figure 4D) in which KCl-induced depolarization rapidly elevated intracellular calcium levels with a gradual decline in intensity shortly thereafter. The synchronized signaling dynamics between cells is particularly evident in the live imaging videos (Supplementary Materials Videos S1 and S2). These results demonstrate functional intracellular signaling within the CO and along the connecting neuronal tract.

### 3.4. Viral Tracing Demonstrates Circuit Integration between COs

**Figure 5:**
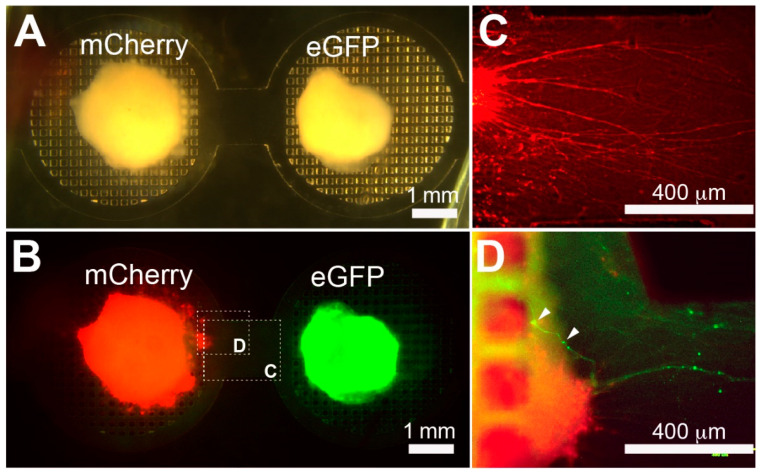
Neurite innervation between viral-transduced human CO slices on grid COCA. (**A**) Brightfield and (**B**) fluorescent images of mCherry-expressing CO slices and eGFP-expressing CO slices cultured on a grid COCA with a 1 mm channel 15 days post-placement. (**C**) Fluorescent images reveal unidirectional neurite outgrowth from the mCherry-expressing CO slice along the connecting channel. (**D**) eGFP-expressing CO neurites innervate a mCherry-expressing CO slice on a grid COCA. White arrows point toward synaptic bouton-like structures.

In order to visualize the contribution of individual neurites between COCA compartments, COs were transduced with either mCherry or eGFP adeno-associated viruses (AAVs). After efficient transduction, the CO slices were cultured on adjacent grid COCA compartments for 15 days prior to imaging with fluorescence microscopy (Figure 5A,B). Figure 5C depicts extensive neurite outgrowth along the connecting channel from the mCherry-labeled CO slice. Overlaying both marker channels revealed eGFP-expressing CO neurites innervating the mCherry-expressing CO slice on the COCA (Figure 5D). There is the presence of synaptic bouton-like structures along the eGFP-labeled neurites, consistent with neuronal morphology. Using this labeling method, it was possible to distinguish respective projections and innervations of CO pairs on the grid COCA.

## 4. Discussion

Brain on a Chip (BoC) systems represent a frontier for modeling the brain in a physiologically relevant setting with key clinical applications in disease modeling and pharmacological screening [15]. Herein, two versions of an in vitro BoC model of three-dimensional neural circuitry between hiPSC-derived CO cultures are presented and their potential to interface with common neuronal characterization techniques, such as calcium imaging, immunolabeling, and viral tracing, are demonstrated. Three-dimensional COs derived from hiPSCs present a natural next step in developing more complex neuronal circuitry models for patient-specific disease modeling due to their genetic specificity, cytoarchitecture, and human context. Other reported studies have demonstrated the formation of major functional axonal tracts between connected COs [27] or fused neural spheroids [26]; however, they are limited in a lack of recirculating flow, spatial constraints which limit overall tissue expansion, and have not demonstrated reliable functional connectivity properties. In this work, a photolithographically defined device termed the COCA was developed, which anchors CO pairs at a distance and enables inter-organoid neuronal tract formation with ample space for expansion and open-top access to characterization metrics.

Given the importance of preserving in vivo dimensions and structure wherever possible, the connecting COCA channel dimensions separate the CO culturing rings at a distance comparable to the millimeter length scale of in vivo cerebral tracts (Figure 1) [33,34]. The diameter of the COCA compartment rings is three-fold larger than the approximate diameter of the COs used in this study (approximately 1000 μm) in order to prevent spatial constraints to tissue growth and permit easier user handling. The open-top configuration of the COCA platform permits media flow around all surfaces of the CO and provides an unconstrained environment as the CO continues to expand both laterally and longitudinally (Figure 2). The connecting channel between the two CO compartments provides a bridge for neurite extension, guiding them to grow unidirectionally toward each side. COs maintained in static culture will naturally fuse and form local neurite connections in random orientations; the COCA’s connecting channel, however, is designed to limit their growth area, thus influencing neurite extension in a unidirectional, rather than stochastic, path. This strategy of applying dimensional patterning to modulate the structural connections of neurons has previously been reported in neural network studies. In this way, the COCA can be likened to the frame or skeleton of the CO culture over which the tissue can expand indefinitely.

The COCA can be readily integrated with biomedical research since the organoids can be manually placed on the device and cultured in an incubator or microscope stage to gather functional data of cellular morphology, viability, growth dynamics, and electrophysiology. Neuronal lineage of generated COs was confirmed via immunostaining and further characterized via visualization of cellular morphology on the devices. Culturing CO bodies or slices on the spiked or grid COCA, respectively, demonstrated extensive neurite growth along the channel and morphological innervation between CO pairs (Figure 3 and Figure 5). Transduction of COs with distinct AAVs facilitated visualization of individual neurite projections and interactions between CO pairs on the COCA (Figure 5). These data illustrated viability and growth on the microfabricated structure, confirming biocompatibility of SU-8 photoresist for cell culture, as previously reported [35], and amenability to standard benchtop microscopy for morphological characterization. Calcium imaging of CO pairs cultured on the COCA demonstrated synchronized calcium ion-mediated signaling within each CO and connecting neuronal tract (Figure 4 and Videos S1 and S2). In addition to calcium sensor imaging, the COCA design and open-top platform are also amenable to morphological and functional analyses such as patch-clamp electrophysiology and whole-mount immunostaining. Collectively, these data show that COs cultured on the COCA generate functional neural tracts, demonstrating the device’s potential to simulate the macro-scale neuronal networks connecting distal brain regions in vivo.

Over the past few years, multiple protocols for generating COs have been published [21,22,30,36,37,38,39], and better protocols continue to be generated [32,40,41,42,43,44]. While COs offer great potential for the study of human-specific phenotypic differences in mental disorders compared to traditional 2D cell culture and animal models, it is important to note key limitations of current human CO systems, specifically an absence of complete cell diversity and lack of reproducibility across cultures [45,46]. Due to the lack of a standardized protocol in the field as well as stochastic extracellular matrix interactions, which affect overall tissue organization, there is inherent variability between organoid cultures even among those generated within the same batch under identical conditions. Secondly, the lack of vascularization within organoid cultures prevents cells in the center of the tissue from receiving sufficient nutrients and oxygen, resulting in the formation of a necrotic core. This compromises the long-term viability of the culture and imposes overall size limitations for organoid cultures, making it difficult to undertake long-term studies of fused organoid cultures. Typically, media diffusion throughout the tissue is enhanced by culturing organoids on orbital shakers or bioreactors, but the issue becomes unavoidable once an organoid grows too large. Given the relatively nascent state of the CO field, optimization of culturing protocols is crucial to overcome these critical caveats and provide more reliable organoid models moving forward. The two presented COCA designs (spiked and grid) and open-top media perfusion platform are compatible with intact or sliced organoid cultures and address the barriers to nutrient and waste exchange seen in previous fused CO cultures [24,47,48,49]. Thus, the developed model system may enable longitudinal investigation of development over time as well as reduced cell culturing expenses and time.

While spiked COCAs adequately secured CO bodies in place, it must be noted that the nature of anchoring the COs onto the compartments may produce unwanted tissue damage in those immediate areas. While overall handling of the cultures and incorporation of COs onto the spiked COCA are performed as gently as possible, the placement may elicit a stress response in the surrounding cell population which could adversely affect their development, intrinsic signaling pathways, and kinetic behavior. The grid COCA circumvents this issue since the tissues are instead gently placed on top. While the open grid structure enables media penetration from beneath the scaffold, areas directly in contact with the grid lines remain inaccessible to the media directly. However, due to the relatively small width of the grid lines in comparison to the overall CO size, it is assumed that the diffusion of media from the open tissue segments is sufficient. Lastly, an important consideration for 3D cultures is to maintain an open platform to permit unconstrained growth space for CO expansion around the device. Constructing organoid models using this key design parameter in addition to a nutrient delivery system to sustain tissue viability will allow researchers to scale up their models and reliably study complex interactions in neural circuitry in vitro.

The reported COCA is microfabricated using standard photolithography materials and processing. As such, device features on transparency masks for LWUV light exposure can be adapted as needed for specific applications. The COCA can be adapted to model specific neural circuits heavily involved in neurodevelopmental processes. The ability to pattern COs into specific lineages, such as thalamic, ventral, and cortical brain regions, makes this a promising application to study a critical circuit in isolation while maintaining relevant physiological cues [22,23,24]. This is critical for the study of mental disorders because it has been recognized that aberrant long-range connectivity across different brain regions contributes to the underlying pathophysiology [4,5,50,51,52,53]. Moreover, the COCA and accompanying PDMS platform are ideal for the integration and study of glial cells in myelination disorders of the central and peripheral nervous systems using oligodendrocytes or Schwann cells, respectively, in which these cells migrate, prune, and wrap around neuronal axons to facilitate synaptic neurotransmission. Importantly, this culturing system can be utilized for pharmacological screening assays, facilitated via continuous perfusion of compounds inside the PDMS chamber. This is relevant for assessing treatment strategies against disorders associated with neuronal tract dysfunction and aberrant connectivity.

## 5. Conclusions

This work combines microfabrication and human iPSC cell culture techniques to describe the fabrication and utility of in vitro culturing platforms to model three-dimensional reciprocal neuronal circuits formed between brain organoids. The scaffold was fabricated using SU-8 photoresist patterning and lift-off strategies with a suspended device design that facilitates increased nutrient delivery around the tissue to combat diffusion limit issues native to in vitro CO cultures. CO bodies or slices can be effectively cultured on the SU-8 scaffold to enable reciprocal tract formation and characterized using common morphological and functional analysis techniques. This work provides a proof-of-concept platform for studying circuitry-based dysfunction in human mental disorders. The use of the described COCA systems has the potential to advance research and development of targeted, patient-specific therapeutic strategies against mental disorders associated with abnormal brain structure and function.

## Figures and Tables

**Table 1:** A summary of the main design parameters of the two COCA versions. Feature length for the spiked and grid COCA represents the length of the individual spikes or square grids, respectively, within each compartment.

Feature	Spiked COCA	Grid COCA
Pattern	Spiked compartments	Grid compartments
Material	SU-8 photoresist	SU-8 photoresist
Thickness	320 μm	320 μm
Feature Length	0.5 mm	0.15 mm

## Data Availability

The data presented in this study are available in this article and supplementary material.

## Supplementary Materials

The following are available online at https://www.mdpi.com/article/10.3390/mi12121574/s1, Video S1: Synchronized neural circuit activity within a CO cultured on compartmentalized spiked COCA for 6 weeks, Video S2: Synchronized neural circuit activity along a connecting CO axonal tract on compartmentalized spiked COCA.
