# Supplementary material for: A Cerebral Organoid Connectivity Apparatus to Model Neuronal Tract Circuitry
Source: Micromachines (Basel). 2021 Dec 17;12(12):1574. doi: 10.3390/mi12121574 (PMC8706388; doi:10.3390/mi12121574)
Supplement: Supplementary file 1 [file micromachines-12-01574-s001.zip › Supplementary Information_v2.pdf]

SUPPLEMENTARY INFORMATION

A Cerebral Organoid Connectivity Apparatus to Model Neuronal Tract Circuitry

Denise A. Robles [1,2], Andrew J. Boreland [2,3], Zhiping P. Pang [2,3,4], Jeffrey D. Zahn [1]

[1]Department of Biomedical Engineering, Rutgers University, 599 Taylor Road, Piscataway, NJ 08854, USA
[2]Child Health Institute of New Jersey, Robert Wood Johnson Medical School, 89 French Street, New Brunswick, NJ 08901, USA
[3]Department of Neuroscience and Cell Biology, 675 Hoes Lane West, Research Tower, Third Floor, Piscataway, NJ 08854, USA
[4]Pediatrics, Robert Wood Johnson Medical School, Rutgers University, One Robert Wood Johnson Place, MEB Third, PO Box 19, New Brunswick, NJ, 08903, USA

Correspondence should be addressed to Jeffrey D. Zahn (jdzahn@soe.rutgers.edu)

TABLE OF CONTENTS

Supplementary Videos

Video S1. Synchronized neural circuit activity within a CO cultured on compartmentalized spiked COCA for 6 weeks.
Video S2. Synchronized neural circuit activity along a connecting CO axonal tract on compartmentalized spiked COCA.

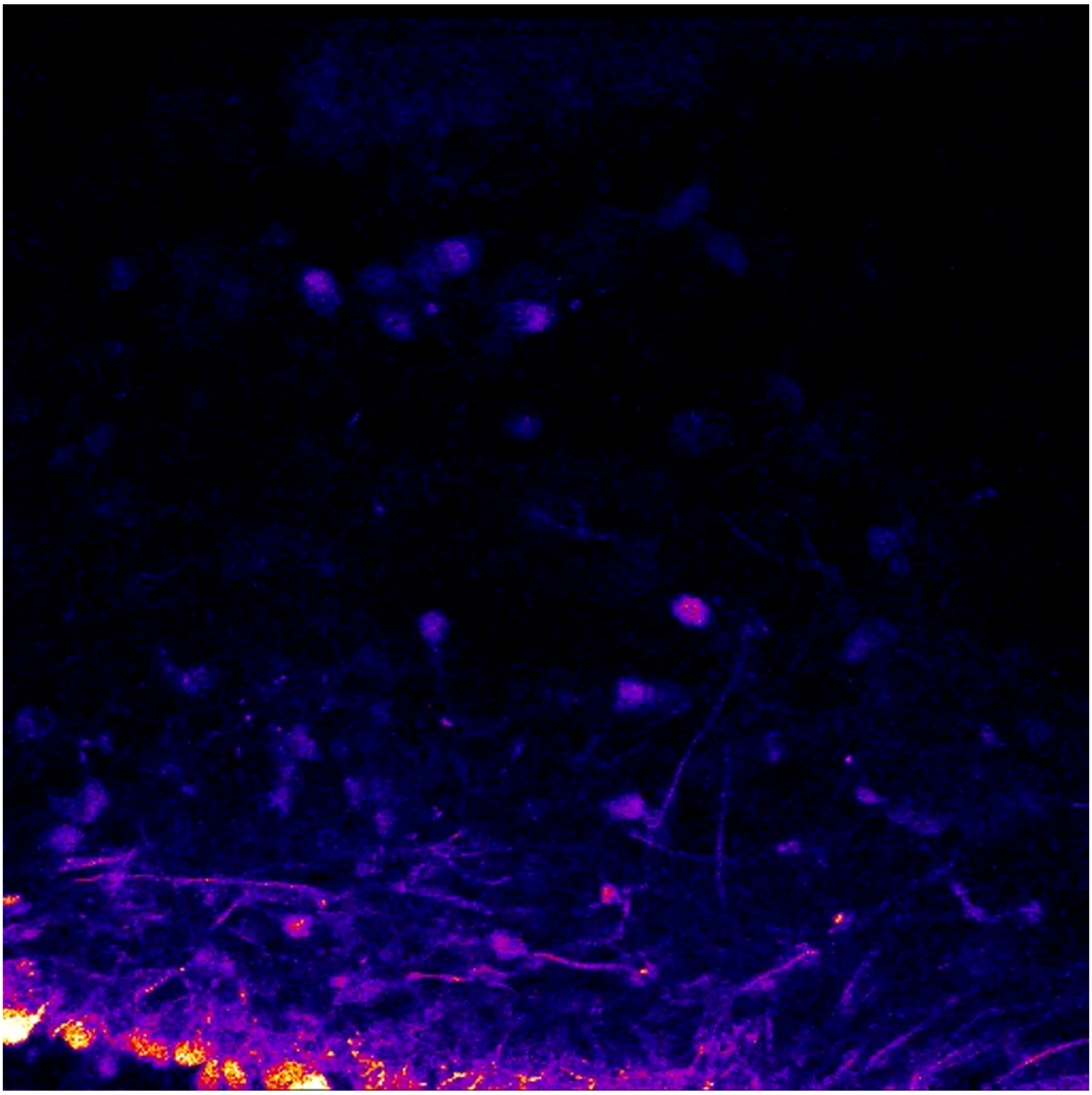

Video S1. Synchronized neural circuit activity within a CO cultured on compartmentalized spiked COCA for 6 weeks. During basal activity, neurons located within each CO side on the COCA demonstrated isolated spontaneous activity, indicating synaptic neurotransmission mediated by local axonal connections. After recording baseline activity, potassium chloride (80 mM, KCl) was added to depolarize all neuronal cells and verify cell viability. KCl addition resulted in a substantial increase in fluorescent intensity across surrounding neurons.

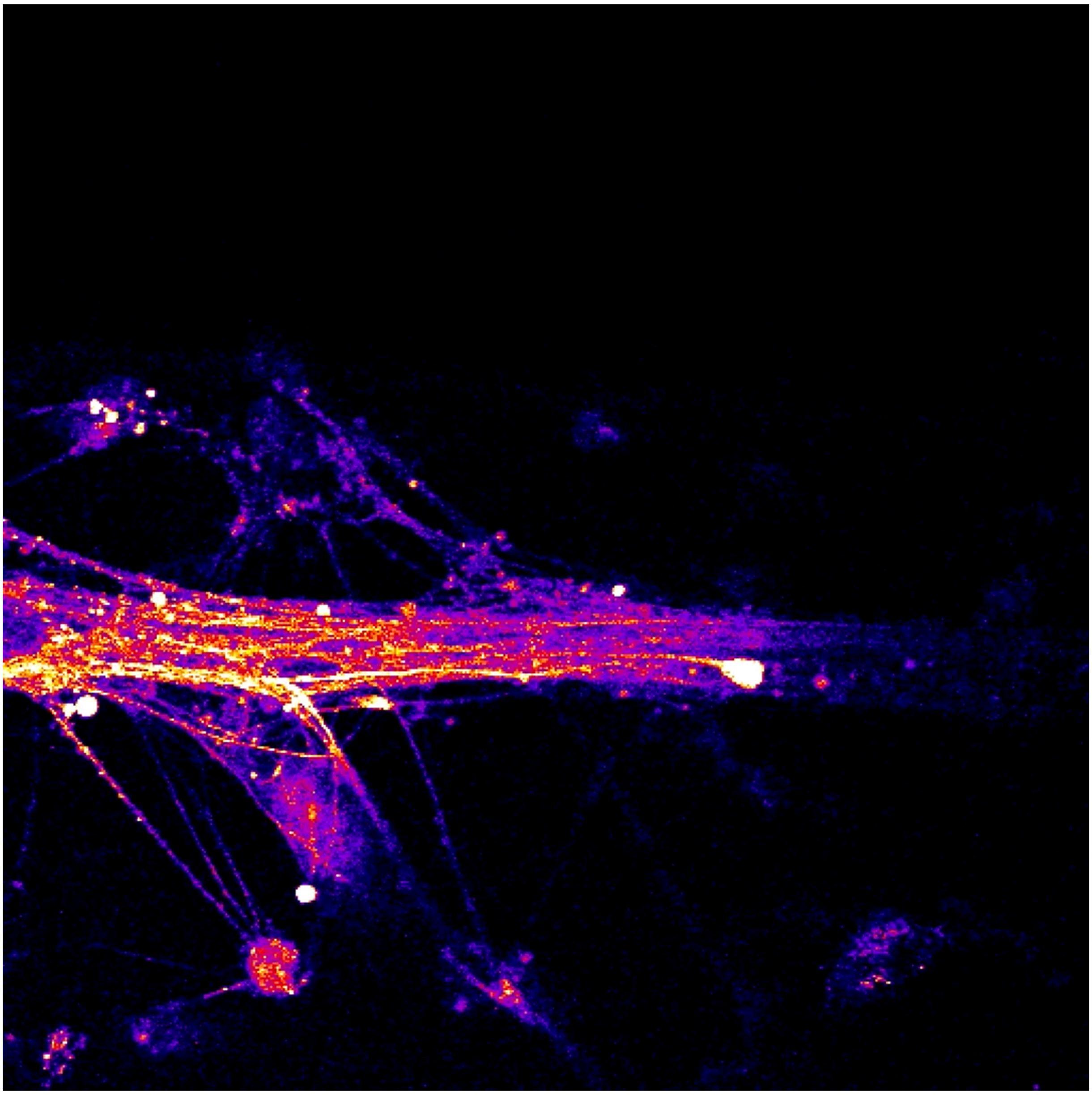

Video S2. Synchronized neural circuit activity along a connecting CO axonal tract on compartmentalized spiked COCA. Neuronal axons located on the connecting channel between COCA compartments demonstrated isolated spontaneous activity during baseline recordings. Potassium chloride (80 mM, KCl) addition elicited a substantial increase in fluorescent intensity in individual axons, indicating functional intracellular signaling along the axonal tract bridging the CO pair.
